# Supplementary material for: Accelerated functional brain aging in pre-clinical familial Alzheimer’s disease
Source: Nat Commun. 2021 Sep 9;12:5346. doi: 10.1038/s41467-021-25492-9 (PMC8429427; doi:10.1038/s41467-021-25492-9)
Supplement: Supplementary file 1 — Supplementary Information [file 41467_2021_25492_MOESM1_ESM.pdf]

Supplementary information

Accelerated functional brain aging in pre-clinical familial Alzheimer's disease

Julie Gonneaud, Alex T. Baria, Alexa Pichet Binette, Brian A. Gordon, Jasmeer P. Chhatwal, Carlos Cruchaga, Mathias Jucker, Johannes Levin, Stephen Salloway, Martin Farlow, Serge Gauthier, Tammie L.S. Benzinger, John C. Morris, Randall J. Bateman, John C.S. Breitner, Judes Poirier, Etienne Vachon-Presseau, and Sylvia Villeneuve, Alzheimer's Disease Neuroimaging Initiative (ADNI), Dominantly Inherited Alzheimer Network (DIAN) Study Group, Pre-symptomatic Evaluation of Experimental or Novel Treatments for Alzheimer's Disease (PREVENT-AD) Research Group

Supplementary Fig. 1
Correlation between the 10 graph metrics used as input in the neural network page 2

Supplementary Fig. 2
Age prediction model with and without ComBat harmonization page 3

Supplementary Table 1
Percentage of frames retained from resting-state fMRI scans in each cohort page 4

Supplementary Table 2
Functional brain parcellation (based on Power and Petersen functional atlas) page 5

Supplementary Table 3
Gene primers in DIAN page 7

Supplementary Table 4
Gene primers in PREVENT-AD page 8

Supplementary Methods
Race/ethnicity of the different cohorts and Estimated years to symptom onset page 9
Small-worldness and resilience calculation page 10

Supplementary Notes page 11

Supplementary References page 16

Supplementary Fig. 1

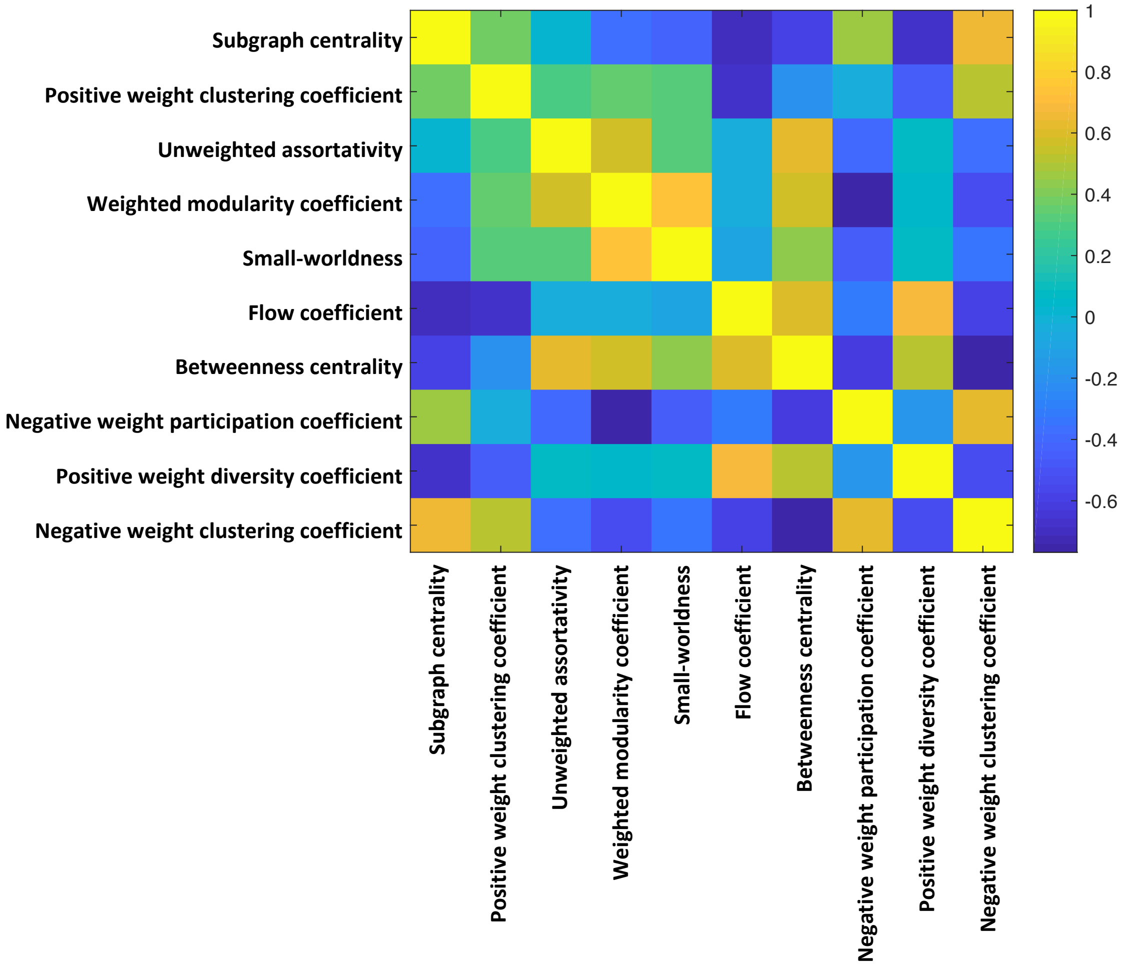

Supplementary Figure 1: Pearson correlations between the 10 graph metrics used as input in the neural network. The color-scale represents r-values; stronger positive correlations being represented by lighter (yellow) colors while darker (blue) colors correspond to stronger negative correlations.

Supplementary Fig. 2

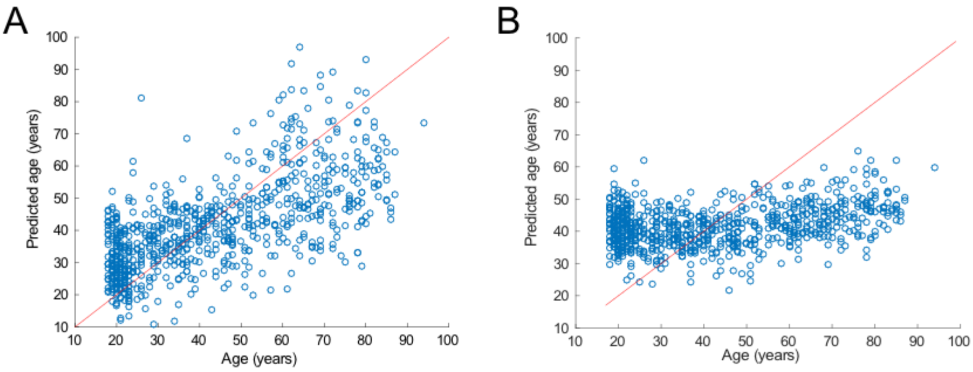

Supplementary Figure 2. Age prediction from support vector machine models using original graph metrics as input (A) and age prediction from support vector machine models using harmonized graph metrics from ComBat as input. (B)

Supplementary Table 1. Percentage of frames retained from resting-state fMRI scans in each cohort

Cohort	Average % frames retained ± SD
CamCAN	86.2 ± 15.5
FCP-Cambridge	100 ± 0
DIAN	93.8 ± 12.0
Prevent-AD	85.0 ± 17.2
ADNI	80.7 ± 15.4
ICBM	96.76 ± 8.0

SD: standard deviation

Supplementary Table 2. Functional brain parcellation (based on Power and Petersen functional atlas)

ROI	MNI space X	Y	Z	Suggested System
13	-7	-52	61	Sensory/somatomotor Hand
14	-14	-18	40	Sensory/somatomotor Hand
15	0,1	-15	47	Sensory/somatomotor Hand
16	9,5	-2	45	Sensory/somatomotor Hand
17	-7	-21	65	Sensory/somatomotor Hand
18	-7	-33	72	Sensory/somatomotor Hand
19	13	-33	75	Sensory/somatomotor Hand
20	-54	-23	43	Sensory/somatomotor Hand
21	29	-17	71	Sensory/somatomotor Hand
22	9,9	-46	73	Sensory/somatomotor Hand
23	-23	-30	72	Sensory/somatomotor Hand
24	-40	-19	54	Sensory/somatomotor Hand
25	29	-39	59	Sensory/somatomotor Hand
26	50	-20	42	Sensory/somatomotor Hand
27	-38	-27	69	Sensory/somatomotor Hand
28	20	-29	60	Sensory/somatomotor Hand
29	44	-8	57	Sensory/somatomotor Hand
30	-29	-43	61	Sensory/somatomotor Hand
31	10	-17	74	Sensory/somatomotor Hand
32	22	-42	69	Sensory/somatomotor Hand
33	-45	-32	47	Sensory/somatomotor Hand
34	-21	-31	61	Sensory/somatomotor Hand
35	-13	-17	75	Sensory/somatomotor Hand
36	42	-20	55	Sensory/somatomotor Hand
37	-38	-15	69	Sensory/somatomotor Hand
38	-16	-46	73	Sensory/somatomotor Hand
39	2,4	-28	60	Sensory/somatomotor Hand
40	3,5	-17	58	Sensory/somatomotor Hand
41	38	-17	45	Sensory/somatomotor Hand
42	-49	-11	35	Sensory/somatomotor Mouth
43	36	-9	14	Sensory/somatomotor Mouth
44	51	-6	32	Sensory/somatomotor Mouth
45	-53	-10	24	Sensory/somatomotor Mouth
46	66	-8	25	Sensory/somatomotor Mouth
47	-3	2,4	53	Cingulo-opercular Task Control
48	54	-28	34	Cingulo-opercular Task Control
49	19	-8	64	Cingulo-opercular Task Control
50	-16	-5	71	Cingulo-opercular Task Control
51	-10	-2	42	Cingulo-opercular Task Control
52	37	0,8	-4	Cingulo-opercular Task Control
53	13	-1	70	Cingulo-opercular Task Control
54	6,5	7,7	51	Cingulo-opercular Task Control
55	-45	0,1	8,8	Cingulo-opercular Task Control
56	49	8,3	-1	Cingulo-opercular Task Control
57	-34	3,3	4,2	Cingulo-opercular Task Control
58	-51	8,3	-2	Cingulo-opercular Task Control
59	-5	18	34	Cingulo-opercular Task Control
60	36	10	1,2	Cingulo-opercular Task Control
61	32	-26	13	Auditory
62	65	-33	20	Auditory
63	58	-16	7,5	Auditory
64	-38	-33	17	Auditory
65	-60	-25	14	Auditory
66	-49	-26	5,2	Auditory
67	43	-23	20	Auditory
68	-50	-34	26	Auditory
69	-53	-22	23	Auditory
70	-55	-9	12	Auditory
71	56	-5	13	Auditory
72	59	-17	29	Auditory
73	-30	-27	12	Auditory

ROI	MNI space X	Y	Z	Suggested System
74	-41	-75	26	Default mode
75	5,6	67	-4	Default mode
76	8,4	48	-15	Default mode
77	-13	-40	0,9	Default mode
78	-18	63	-9	Default mode
79	-46	-61	21	Default mode
80	43	-72	28	Default mode
81	-44	12	-34	Default mode
82	46	16	-30	Default mode
86	-44	-65	35	Default mode
87	-39	-75	44	Default mode
88	-7	-55	27	Default mode
89	5,9	-59	35	Default mode
90	-11	-56	16	Default mode
91	-3	-49	13	Default mode
92	7,9	-48	31	Default mode
93	15	-63	26	Default mode
94	-2	-37	44	Default mode
95	11	-54	17	Default mode
96	52	-59	36	Default mode
97	23	33	48	Default mode
98	-10	39	52	Default mode
99	-16	29	53	Default mode
100	-35	20	51	Default mode
101	22	39	39	Default mode
102	13	55	38	Default mode
103	-10	55	39	Default mode
104	-20	45	39	Default mode
105	5,9	54	16	Default mode
106	6,1	64	22	Default mode
107	-7	51	-1	Default mode
108	8,8	54	3,5	Default mode
109	-3	44	-9	Default mode
110	7,5	42	-5	Default mode
111	-11	45	7,6	Default mode
112	-2	38	36	Default mode
113	-3	42	16	Default mode
114	-20	64	19	Default mode
115	-8	48	23	Default mode
117	-56	-13	-10	Default mode
118	-58	-30	-4	Default mode
119	65	-31	-9	Default mode
120	-68	-41	-5	Default mode
121	13	30	59	Default mode
122	12	36	20	Default mode
123	52	-2	-16	Default mode
124	-26	-40	-8	Default mode
125	27	-37	-13	Default mode
126	-34	-38	-16	Default mode
127	28	-77	-32	Default mode
128	52	6,8	-30	Default mode
129	-53	2,6	-27	Default mode
130	47	-50	29	Default mode
131	-49	-42	0,8	Default mode
133	-2	-35	31	Memory retrieval
134	-7	-71	42	Memory retrieval
135	11	-66	42	Memory retrieval
136	4,2	-48	51	Memory retrieval
137	-46	31	-13	Default mode

138	-10	11	67	Ventral attention
139	49	35	-12	Default mode

	**MNI space**			**Suggested System**
ROI	**X**	**Y**	**Z**	
143	18	-47	-10	Visual
144	40	-72	14	Visual
145	8,5	-72	11	Visual
146	-8	-81	7,4	Visual
147	-28	-79	19	Visual
148	20	-66	1,7	Visual
149	-24	-91	19	Visual
150	27	-59	-9	Visual
151	-15	-72	-8	Visual
152	-18	-68	4,8	Visual
153	43	-78	-12	Visual
154	-47	-76	-10	Visual
155	-14	-91	31	Visual
156	15	-87	37	Visual
157	29	-77	25	Visual
158	20	-86	-2	Visual
159	15	-77	31	Visual
160	-16	-52	-1	Visual
161	42	-66	-8	Visual
162	24	-87	24	Visual
163	5,6	-72	24	Visual
164	-42	-74	0,4	Visual
165	26	-79	-16	Visual
166	-16	-77	34	Visual
167	-3	-81	21	Visual
168	-40	-88	-6	Visual
169	37	-84	13	Visual
170	6,2	-81	6,1	Visual
171	-26	-90	3,1	Visual
172	-33	-79	-13	Visual
173	37	-81	1,2	Visual
174	-44	1,8	46	Fronto-parietal Task Control
175	48	25	27	Fronto-parietal Task Control
176	-47	11	23	Fronto-parietal Task Control
177	-53	-49	43	Fronto-parietal Task Control
178	-23	11	64	Fronto-parietal Task Control
179	58	-53	-14	Fronto-parietal Task Control
180	24	45	-15	Fronto-parietal Task Control
181	34	54	-13	Fronto-parietal Task Control
186	47	9,9	33	Fronto-parietal Task Control
187	-41	5,8	33	Fronto-parietal Task Control
188	-42	38	21	Fronto-parietal Task Control
189	38	43	15	Fronto-parietal Task Control
190	49	-42	45	Fronto-parietal Task Control
191	-28	-58	48	Fronto-parietal Task Control
192	44	-53	47	Fronto-parietal Task Control
193	32	14	56	Fronto-parietal Task Control
194	37	-65	40	Fronto-parietal Task Control
195	-42	-55	45	Fronto-parietal Task Control
196	40	18	40	Fronto-parietal Task Control
197	-34	55	4,4	Fronto-parietal Task Control
198	-42	45	-2	Fronto-parietal Task Control
199	33	-53	44	Fronto-parietal Task Control
200	43	49	-2	Fronto-parietal Task Control
201	-42	25	30	Fronto-parietal Task Control
202	-3	26	44	Fronto-parietal Task Control
203	11	-39	50	Salience
204	55	-45	37	Salience
205	42	-0	47	Salience
206	31	33	26	Salience
207	48	22	9,7	Salience
208	-35	20	0,1	Salience
209	36	22	2,6	Salience
210	37	32	-2	Salience

	**MNI space**			**Suggested System**
ROI	**X**	**Y**	**Z**	
211	34	16	-8	Salience
212	-11	26	25	Salience
213	-1	15	44	Salience
214	-28	52	21	Salience
215	-0	30	27	Salience
216	5,2	23	37	Salience
217	10	22	27	Salience
218	31	56	14	Salience
219	26	50	27	Salience
220	-39	51	17	Salience
221	1,8	-24	30	Memory retrieval
222	6,3	-24	-0	Subcortical
223	-2	-13	12	Subcortical
224	-10	-18	7	Subcortical
225	12	-17	7,5	Subcortical
226	-5	-28	-4	Subcortical
227	-22	7,5	-5	Subcortical
228	-15	3,6	8	Subcortical
229	31	-14	1,7	Subcortical
230	23	10	1,5	Subcortical
231	29	0,8	4	Subcortical
232	-31	-11	-0	Subcortical
233	15	4,9	7,2	Subcortical
234	8,6	-4	5,8	Subcortical
235	54	-43	22	Ventral attention
236	-56	-50	9,9	Ventral attention
237	-55	-40	14	Ventral attention
238	52	-33	7,6	Ventral attention
239	51	-29	-4	Ventral attention
240	56	-46	11	Ventral attention
241	53	33	0,6	Ventral attention
242	-49	25	-1	Ventral attention
251	9,6	-62	61	Dorsal attention
252	-52	-63	5,3	Dorsal attention
255	47	-30	49	Sensory/somatomotor Hand
256	22	-65	48	Dorsal attention
257	46	-59	3,9	Dorsal attention
258	25	-58	60	Dorsal attention
259	-33	-46	47	Dorsal attention
260	-27	-71	37	Dorsal attention
261	-32	-1	54	Dorsal attention
262	-42	-60	-9	Dorsal attention
263	-17	-59	64	Dorsal attention
264	29	-5	54	Dorsal attention
265	-10	14	-2	Limbic
266	10	14	-2	Limbic
267	-22	-2	-22	Limbic
268	26	-2	-22	Limbic
269	-24	-14	-18	Limbic
270	26	-14	-18	Limbic
271	-28	-34	-6	Limbic
272	30	-34	-6	Limbic

Supplementary Table 3: Gene primers in DIAN

Gene	Ensembl_Transcript_ID	Exon	Direction	Sequence
PSEN2	ENST00000366783.3	4	Forward	GACAGGCATCTCTTGGAAGC
		4	Reverse	CATCAGGGAATGAATGTCTGG
		5	Forward	ACTTCTCATTTCTGGTTCCA
		5	Reverse	TAGGTCACAATCCAGGAGG
		6	Forward	ACTCCATCAGGGCAGCAT
		6	Reverse	AAAAATCTGGGTCTATTTTCCTCT
		8	Forward	GTTGGGACTGAATGGTGGTA
		8	Reverse	CCCTCTGTTTTACAAAGGCG
PSEN1	ENST00000324501.5	4	Forward	AACTCATAGTGACGGGTCTG
		4	Reverse	GTAATAACCCCTCGCTCTCT
		5	Forward	TTGGTGAGTTGGGGAAA
		5	Reverse	CACAGTGAGGAGGAAGAAAA
		6	Forward	CGACAAAGTGAGACCCTGT
		6	Reverse	AGTACATGGCTTTAAATGATAGCT
		7	Forward	ATGTTTGGGAGCCATCA
		7	Reverse	CCAGCCGAAATCTTCAA
		8	Forward	TCACCTGCCATTTATTTCA
		8	Reverse	CAGGAATGCTGTGCATTTA
		9	Forward	CTGCTAAAACCAAAGAGAACC
		9	Reverse	TGTATTTACTGGGCATTATCATAG
		11	Forward	AAAACACAGCTGAAGCCTAA
		11	Reverse	GCTCCTCAGATAGCTGGAAT
		12	Forward	TCCAGATTGAATGAACGTCT
		12	Reverse	TGGAAGGAAGCTGCAAA
APP	ENST00000346798.3	7	Forward	ATGCTGCCTAATAAACCAGTCC
		7	Reverse	TCCCAAGAACCAGGAAAATCAA
		16	Forward	GGTTTCCCTTACCCTTTCATTT
		16	Reverse	TCAGCCTAGCCTATTTATTTTCT
		17	Forward	TGAAACTTTTTATATAACCTCATCCAA
		17	Reverse	CATGGAAGCACACTGATTCG

The table is annotated based on Ensembl version 75 in genome build GRCh37

Supplementary Table 4: Gene primers in PREVENT-AD

Gene	Variant	Analyses	Sequence
APOE	rs429358	amplification forward	5'-ACGGCTGTCCAAGGAGCTG-3'
		amplification reverse biotinylated	5'-CACCTCGCCGCGGTACTG-3'
		sequencing	5'-CGGACATGGAGGACG-3'
	rs7412	amplification forward	5'-CTCCGCGATGCCGATGAC-3'
		amplification reverse biotinylated	5'-CCCCGGCCTGGTACACTG-3'
		sequencing	5'-CGATGACCTGCAGAAG-3'

Supplementary Methods

Race/ethnicity from the different cohorts

DIAN: The sample mainly identified as non-Hispanic/White, both for mutation non-carriers (90% from the training set and 100% from the test set) and mutation carriers (80% from the test set).
The ten remaining mutation non-carriers (all from the training set) identified as Hispanic/White (n=4), Hispanic with no further specification (n=2), non-Hispanic/Middle Eastern (n=1) or non-Hispanic/Aboriginal (n=2). Regarding the 24 mutation carriers with a different race/ethnicity, they identified themselves as Hispanic/White (n=10), Hispanic with no further specification (n=6), non-Hispanic/Middle Eastern-North Africa (n=2), Aboriginal (n=1), native Hawaiian or other pacific islanders (n=3), Hispanic/Black or African American (n=1) and non-Hispanic/Asian (n=1).

PREVENT-AD: The sample was mainly White/Caucasian with the exception of 4 participants (2 Hispanics, 1 Haitian and 1 unspecified).

ADNI: The sample mainly identified as non-Hispanic/White (83% of those included in the training set and 93% of those included in the test set), with the exception of 6 subjects (1 Hispanic/White, 1 unknow ethnicity/White and 3 non-Hispanic/not White [1 Black, 1 with more than one race and 1 unknown] in the training set, and 1 Hispanic/White in the test set).

FCP-Cambridge, CamCAN and ICBM: Participants from the FCP-Cambridge were recruited from the Cambridge (MA, USA) area, CamCAN is a population-based cohort recruited within the Cambridge City (UK) area (excluding term-time residents of colleges and universities) and the ICBM cohort was recruited in the Montreal (QC, Canada) area; however further demographic information, including specific information on race/ethnicity, was not provided for these cohorts.

Estimated years to symptom onset

Estimated expected years to symptom onset (EYO) was computed in the two cohorts by subtracting each participant's age at assessment from his/her parent's age at symptom onset. In DIAN, the parental age at onset was determined using semi-structured interview in which family members were asked about the age of first progressive cognitive decline.[1] In PREVENT-

AD, EYO was calculated using the age of the parent at which the family observed significant cognitive/memory changes, as reported by the participant during the medical interview.[2–4]

We conducted partial correlations between EYO and the predicted age difference (PAD), controlling for the influence of chronological age, in DIAN and PREVENT-AD.

Calculation of small-worldness and resilience

For a thresholded correlation matrix G, small-worldness was calculated as Supplementary Equation 1:

$$\text{small-worldness} = [(\text{clustering}_G / \text{clustering}_{\text{random}}) / (\text{efficiency}_{\text{random}} / \text{efficiency}_G)] \qquad (1)$$

in which clustering is the clustering coefficient, and indicates the extent to which nodes are clustered together. The efficiency indicates the average of the inverse path length between nodes of the matrix. The subscript random indicates when these measures are taken on randomly scrambled matrices with preserved degree count for each node in G, and were generated using the function randmio_und. Random clustering coefficient and efficiency were averaged over 100 random matrices, generated for each scan.

Resilience is a measure of the robustness of network G as node hubs are removed. Networks with scale-free properties *(i.e.* node degree probabilities follow a power-law distribution) are resilient to random attacks and can be described as Supplementary Equation 2:

$$p(k) \alpha k^{-y} \qquad (2)$$

where $p(k)$ is the probability of a node having a degree of k (or k total connections), and y is an exponent. On a log-log scale this probability distribution is linear, and thus resilience of G can be estimated as the negative slope of the degree distribution.

Supplementary Notes. DIAN Study Group

Last Name	First	Institution	Affiliation	Core	Role	Email address
Allegri	Ricardo	FLENI	FLENI Institute of Neurological Research (Fundacion para la Lucha contra las Enfermedades Neurologicas de la Infancia)	N/A	PI	rallegri@fleni.org.ar
Bateman	Randy	WU	Washington University in St. Louis School of Medicine	Admin	**Core Leader/PI/Chair**	batemanr@wustl.edu
Bechara	Jacob	Sydney	Neuroscience Research Australia	N/A	Site Leader	j.bechara@neura.edu.au
Benzinger	Tammie	WU	Washington University in St. Louis School of Medicine	Imaging	**Core Leader**	benzingert@wustl.edu
Berman	Sarah	Pitt	University of Pittsburgh	N/A	PI	bermans@upmc.edu
Bodge	Courtney	Butler	Brown University-Butler Hospital	N/A	Site Coordinator	Cbodge@Butler.org
Brandon	Susan	WU	Washington University in St. Louis School of Medicine	Admin / Clincal	Core Personnel	brandons@wustl.edu
Brooks	William (Bill)	Sydney	Neuroscience Research Australia	N/A	Site Coordinator	w.brooks@NeuRA.edu.au
Buck	Jill	IU	Indiana University	N/A	Site Coordinator	jilmbuck@iu.edu
Buckles	Virginia	WU	Washington University in St. Louis School of Medicine	Admin	Core Personnel	bucklesv@wustl.edu
Chea	Sochenda	Mayo	Mayo Clinic Jacksonville	N/A	Site Coordinator	chea.sochenda@mayo.edu
Chhatwal	Jasmeer	BWH	Brigham and Women's Hospital–Massachusetts General Hospital	N/A	PI	Chhatwal.Jasmeer@mgh.harvard.edu
Chrem	Patricio	FLENI	FLENI Institute of Neurological Research (Fundacion para la Lucha contra las Enfermedades Neurologicas de la Infancia)	N/A	Site Coordinator	pchremmendez@fleni.org.ar
Chui	Helena	USC	University of Southern California	N/A	PI	helena.chui@med.usc.edu
Cinco	Jake	UCL	University College London	N/A	Site Coordinator	jcinco@nhs.net
Cruchaga	Carlos	WU	Washington University in St. Louis School of Medicine	Genetics	**Core Co-Leader**	cruchagac@wustl.edu
Donahue	Tamara	WU	Washington University in St. Louis School of Medicine	N/A	Site Coordinator	tammie@wustl.edu
Douglas	Jane	UCL	University College London	N/A	Site Coordinator	jdouglas@dementia.ion.ucl.ac.uk

Last Name	First	Institution	Affiliation	Core	Role	Email address
Edigo	Noelia	FLENI	FLENI Institute of Neurological Research (Fundacion para la Lucha contra las Enfermedades Neurologicas de la Infancia)	N/A	Site Coordinator	negido@fleni.org.ar
Erekin-Taner	Nilufer	Mayo	Mayo Clinic Jacksonville	N/A	*sub-I*	taner.nilufer@mayo.edu
Fagan	Anne	WU	Washington University in St. Louis School of Medicine	Biomarker	**Core Leader**	fagana@wustl.edu
Farlow	Marty	IU	Indiana University	N/A	PI	mfarlow@iupui.edu
Fitzpatrick	Colleen	BWH	Brigham and Women's Hospital-Massachusetts	N/A	Site Co-Coordinator	cdfitzpatrick@bwh.harvard.edu
Flynn	Gigi	WU	Washington University in St. Louis School of Medicine	Admin / Clinical	Core Personnel	flynng@wustl.edu
Fox	Nick	UCL	University College London	N/A	PI	nfox@dementia.ion.ucl.ac.uk
Franklin	Erin	WU	Washington University in St. Louis School of Medicine	Neuropath	Core Coordinator	efranklin@wustl.edu
Fujii	Hisako	Japan	Osaka City University	N/A	Assistant/Coord	hfujii@med.osaka-cu.ac.jp
Gant	Cortaiga	WU	Washington University in St. Louis School of Medicine	Admin / Clinical	Core Personnel	cortaiga.gant@wustl.edu
Gardener	Samanth a	Perth	Edith Cowan University, Perth	N/A	Site Coordinator	s.gardener@ecu.edu.au
Ghetti	Bernardi no	IU	Indiana University	N/A	*sub-I*	bghetti@iupui.edu
Goate	Alison	Icahn NY	Icahn School of Medicine at Mount Sinai	Genetics	**Core Co-Leader**	alison.goate@mssm.edu
Goldman	Jill	CU	Columbia University	N/A	Genetics Ethics	JG2673@cumc.columbia.edu
Gordon	Brian	WU	Washington University in St. Louis School of Medicine	Imaging	Core Personnel	bagordon@wustl.edu
Graff-Radford	Neill	Mayo	Mayo Clinic Jacksonville	N/A	PI	graffradford.neill@mayo.edu
Gray	Julia	WU	Washington University in St. Louis School of Medicine	Biomarker	Core Personnel	gray@wustl.edu
Groves	Alexande r	WU	Washington University in St. Louis School of Medicine	Biomarker	Core Coordinator	amgroves@wustl.edu
Hassenstab	Jason	WU	Washington University in St. Louis School of Medicine	Clinical	Core Personnel	hassenstabj@wustl.edu
Hoechst-Swisher	Laura	WU	Washington University in St. Louis School of Medicine	Admin / Clinical	Core Coordinator	goodl@wustl.edu

Last Name	First	Institution	Affiliation	Core	Role	Email address
Holtzman	David	WU	Washington University in St. Louis School of Medicine	N/A	Associate Director	holtzman@wustl.edu
Hornbeck	Russ	WU	Washington University in St. Louis School of Medicine	Imaging	Core Coordinator	russ@wustl.edu
Houeland DiBari	Siri	Munich	German Center for Neurodegenerative Diseases (DZNE) Munich	N/A	Site Coordinator	Siri.HouelandDiBari@dzne.de
Ikeuchi	Takeshi	Niigata	Niigata University	N/A	*Site Leader*	ikeuchi@bri.niigata-u.ac.jp
Ikonomovic	Snezana	Pitt	University of Pittsburgh	N/A	Site Coordinator	ikonomovics@upmc.edu
Jack	Clifford	Mayo	Mayo Clinic Jacksonville	MRI QC	Vendor MRI QC	jack.clifford@mayo.edu
Jerome	Gina	WU	Washington University in St. Louis School of Medicine	Biomarker	Core Coordinator	ginajerome@wustl.edu
Jucker	Mathias	Tubingen	German Center for Neurodegenerative Diseases (DZNE) Tubingen	N/A	PI	mathias.jucker@uni-tuebingen.de
Karch	Celeste	WU	Washington University in St. Louis School of Medicine	Administrative	Core Personnel	karchc@wustl.edu
Kasuga	Kensaku	Niigata	Niigata University	N/A	Site Coordinator	ken39@bri.niigata-u.ac.jp
Kawarabayashi	Takeshi	Hirosaki	Hirosaki University	N/A	Clinician	tkawara@hirosaki-u.ac.jp
Klunk	William (Bill)	Pitt	University of Pittsburgh	N/A	sub-I	klunkwe@gmail.com
Koeppe	Robert	U of Michigan	University of Michigan	PET QC	Vendor PET QC	koeppe@umich.edu
Kuder-Buletta	Elke	Tubingen	German Center for Neurodegenerative Diseases (DZNE) Tubingen	N/A	Site Coordinator	elke.buletta@med.uni-tuebingen.de
Laske	Christoph	Tubingen	German Center for Neurodegenerative Diseases (DZNE) Tubingen	N/A	*sub-I*	christoph.laske@med.uni-tuebingen.de
Lee	Jae-Hong	Korea	Asan Medical Center	N/A	PI	jhlee@amc.seoul.kr
Levin	Johannes	Munich	German Center for Neurodegenerative Diseases (DZNE) Munich	N/A	PI	Johannes.Levin@med.uni-muenchen.de
Martins	Ralph	Perth	Edith Cowan University	N/A	PI	r.martins@ecu.edu.au
Mason	Neal Scott	UPMC	University of Pittsburgh Medical Center	PIB QC	Vendor PIB QC	masonss@upmc.edu
Masters	Colin	Melb	University of Melbourne	N/A	PI - former	c.masters@unimelb.edu.au

Last Name	First	Institution	Affiliation	Core	Role	Email address
Maue-Dreyfus	Denise	WU	Washington University in St. Louis School of Medicine	Clinical	Core Personnel	dmdreyfu@wustl.edu
McDade	Eric	WU	Washington University in St. Louis School of Medicine	Clinical	**Core Leader Assoc**	ericmcdade@wustl.edu
Mori	Hiroshi	Japan	Osaka City University	N/A	PI	mori@med.osaka-cu.ac.jp
Morris	John	WU	Washington University in St. Louis School of Medicine	Clinical	**Core Leader**	jcmorris@wustl.edu
Nagamatsu	Akem	Tokyo	Tokyo University	N/A	Site Coordinator	akm77-tky@umin.ac.jp
Neimeyer	Katie	CU	Columbia University	N/A	Site Coordinator	kn2416@cumc.columbia.edu
Noble	James	CU	Columbia University	N/A	PI	jn2054@columbia.edu
Norton	Joanne	WU	Washington University in St. Louis School of Medicine	Genetics	Core Coordinator	nortonj@wustl.edu
Perrin	Richard	WU	Washington University in St. Louis School of Medicine	Neuropath	**Core Leader**	rperrin@wustl.edu
Raichle	Marc	WU	Washington University in St. Louis School of Medicine	Imaging	Core Personnel	mraichle@wustl.edu
Renton	Alan	Icahn NY	Icahn School of Medicine at Mount Sinai	Genetics	Core Personnel	alan.renton@mssm.edu
Ringman	John	USC	University of Southern California	N/A	*sub-I*	john.ringman@med.usc.edu
Roh	Jee Hoon	Korea	Asan Medical Center	N/A	*sub-I*	roh@amc.seoul.kr
Salloway	Stephen	Butler	Brown University-Butler Hospital	N/A	PI	SSalloway@Butler.org
Schofield	Peter	Sydney	Neuroscience Research Australia	N/A	PI	p.schofield@neura.edu.au
Shimada	Hiroyuki	Osaka	Osaka City University	N/A	*Site Leader*	h.shimada@med.osaka-cu.ac.jp
Sigurdson	Wendy	WU	Washington University in St. Louis School of Medicine	N/A	Site Coordinator	sigurdsonw@wustl.edu
Sohrabi	Hamid	Perth	Edith Cowan University	N/A	Site Coordinator	h.sohrabi@ecu.edu.au
Sparks	Paige	BWH	Brigham and Women's Hospital-Massachusetts	N/A	Site Coordinator	kpsparks@bwh.harvard.edu
Suzuki	Kazushi	Tokyo	Tokyo University	N/A	*Site Leader*	kazusuzuki-tky@umin.ac.jp
Taddei	Kevin	Perth	Edith Cowan University	N/A	Site Coordinator	k.taddei@ecu.edu.au
Wang	Peter	WU	Washington University in St. Louis School of Medicine	Biostat	Core Coordinator	guoqiao@wustl.edu
Xiong	Chengjie	WU	Washington University in St. Louis School of Medicine	Biostat	**Core Leader**	chengjie@wustl.edu

Last Name	First	Institution	Affiliation	Core	Role	Email address
Xu	Xiong	WU	Washington University in St. Louis School of Medicine	Biostat	Core Personnel	xxu@wustl.edu
Levey	Allan	Emory	Emory University School of Medicine	N/A	Project Leader	alevey@emory.edu

Supplementary References

1. Bateman, R. J. *et al.* Clinical and biomarker changes in dominantly inherited Alzheimer's disease. *N. Engl. J. Med.* **367**, 795–804 (2012).
2. Villeneuve, S. *et al.* Proximity to Parental Symptom Onset and Amyloid-β Burden in Sporadic Alzheimer Disease. *JAMA Neurol* (2018) doi:10.1001/jamaneurol.2017.5135.
3. Vogel, J. W. *et al.* Brain properties predict proximity to symptom onset in sporadic Alzheimer's disease. *Brain* (2018) doi:10.1093/brain/awy093.
4. Gonneaud, J. *et al.* Association of education with Aβ burden in preclinical familial and sporadic Alzheimer disease. *Neurology* **95**, e1554–e1564 (2020).
